# Evaluation and selection of potent fluorescent immunosensors by combining fluorescent peptide and nanobodies displayed on yeast surface

**DOI:** 10.1038/s41598-021-02022-7

**Published:** 2021-11-19

**Authors:** Akihito Inoue, Takanobu Yasuda, Bo Zhu, Tetsuya Kitaguchi, Akikazu Murakami, Hiroshi Ueda

**Affiliations:** 1 Graduate School of Life Science and Technology, Tokyo Institute of Technology, 4259-R1-18 Nagatsuta-cho, Midori-ku, Yokohama, Kanagawa 226-8503 Japan; 2 Laboratory for Chemistry and Life Science, Institute of Innovative Research, Tokyo Institute of Technology, Nagatsuta-cho, Yokohama, Kanagawa 226-8503 Japan; 3 Department of Oral Microbiology, Graduate School of Biomedical Sciences, Tokushima University, 3-18-15 Kuramoto, Tokushima, 770-8504 Japan; 4 RePHAGEN Co., Ltd., Uruma, Okinawa 904-2234 Japan

**Keywords:** Assay systems, Applied immunology, Analytical biochemistry, Immunological techniques, Antibody isolation and purification

## Abstract

Quenchbody (Q-body) is a quench-based fluorescent immunosensor labeled with fluorescent dye(s) near the antigen-binding site of an antibody. Q-bodies can detect a range of target molecules rapidly and directly. However, because Q-bodies show different antigen responses depending on the antibody used, time-consuming optimization of the Q-body structure is often necessary, and a high-throughput screening method for discriminating and selecting good Q-bodies is required. Here, we aimed to develop a molecular display method of nanobody-based “mini Q-bodies” by combining yeast surface display and coiled-coil forming E4/K4 peptide-based fluorescence labeling. As a result, the yeast-displayed mini Q-body recognizing the anti-cancer agent methotrexate (MTX) showed significant quenching and MTX-dependent dequenching on cells. To demonstrate the applicability of the developed method to select highly responsive mini Q-bodies, a small nanobody library consisting of 30 variants that recognize human serum albumin was used as a model. The best variant, showing a 2.4-fold signal increase, was obtained through selection by flow cytometry. Furthermore, the same nanobody prepared from *Escherichia coli* also worked as a mini Q-body after dye labeling. The described approach will be applied to quickly obtain well-behaved Q-bodies and other fluorescent biosensors for various targets through directed evolutionary approaches.

## Introduction

Immunoassays have become increasingly important in analytical and clinical research. These are based on the specificity and affinity of antigen–antibody reactions, which offer crucial benefits for the detection of small amounts of target antigens such as hazards in food samples^1^, biomarkers in body fluids^2^, and contaminating substances in environmental samples^3^. To date, many types of immunoassay methods have been developed. For example, enzyme-linked immunosorbent assays (ELISAs) are generally used in clinical diagnosis since they were first reported in 1972^4^. Despite their high sensitivity, the requirement of multiple reaction steps makes the process time-consuming and technically complex, which could be a drawback in point-of-care testing (POCT) applications. Although, several immunoassays had been re-designed for POCT devices^5–7^, few researchers focused on engineering antibody or transducing molecules to develop novel immunosensing principle^8,9^.

“Quenchbody (Q-body)” is an antibody-based novel fluorescent immunosensor, in which a fluorophore is labeled near the antigen-binding site of an antibody fragment such as a single-chain variable region (scFv) or Fab fragment^10^. Recently, an anti-methotrexate (MTX) nanobody (V_HH_) has been proven to become a smaller Q-body (mini Q-body)^11^. We also found that several V_H_ monodomains can be converted to mini Q-bodies (Banwait et al., in preparation). This has led to the broadening of the field of application, including their application in POCT^12^ or intracellular imaging^13^. The Q-body works on the principle of antigen-dependent removal of the quenching effect on a fluorophore that has been quenched by intrinsic tryptophan (Trp) residues of an antibody fragment^14^. Because it does not require bound/free separation and detects antigens in a noncompetitive manner, the Q-body-based assay is simple, rapid, and exhibits a relatively high sensitivity. In particular, small haptens can be detected with a sensitivity superior to that of other competitive immunoassays^10^. Recently, Q-body has been successfully converted to a bioluminescent sensor (BRET Q-body)^15^, which achieved a higher sensitivity and enhanced response than conventional Q-body. By just mixing the BRET Q-body and luminescent substrate with sample, the assay completes within 5 min. Moreover, it does not need a light source, allowing visual observation of the color change and easier integration to a smartphone-based device.

To date, the Q-body has successfully detected various targets from small haptens to large molecules, depending on the aforementioned mechanism by rational design^16^. Compared with other fluorescent immunosensor approaches such as Scaffold Conjugated to Environment Sensitive Fluorophore (SuCESsFul) biosensors^17^, which is based on the interaction between protein antigen and environment-sensitive fluorophore, Q-body can also detect small molecules and boasts more choices for optimization; variety of linker length, type of fluorescent dye, and labeling positions^16^. However, the response of the Q-body depends on the antibody used, which means that if the antibody is not suitable for the Q-body, the above optimization cannot work. Therefore, the development of a combinatorial approach involving larger library construction and high-throughput screening methods to identify potent antibodies for Q-bodies is in demand.

Here, we aimed to develop a novel combinatorial method to select Q-bodies that show high fluorescent responses from a large antibody library by combining two key technologies, yeast surface display and coiled-coil interaction. Yeast surface display is a well-established selection method for functional proteins. The protein of interest (POI), such as an antibody fragment, is displayed on the yeast cell surface by fusing with a cell wall protein and screened for superior properties, such as higher antigen-binding activity, using a high-throughput method such as flow cytometry. Since it was first used for screening scFv antibody fragments in 1997^18^, its application has ranged from the affinity maturation of antibodies to directed evolution of the enzyme^19^. On the other hand, coiled-coil forming peptides are a pair of helical peptides that are rationally designed to show a high affinity between two or more^20^. As the proteins tethered with these peptides spontaneously form a multimer simply by mixing them in a mild environment, this technology has been used to rapidly assemble two or more proteins in vitro or in vivo^21^. Recently, an E4/K4 peptide pair was used to introduce fluorescent dye into the N-terminal region of the Q-bodies (Coiled Q-bodies); Fab and nanobody-based Q-bodies were constructed with comparable responses to conventional Q-bodies using an amber codon or Cys residue as a labeling strategy^22^. Therefore, when the yeast-displayed antibody is labeled using the E4/K4 peptide pair, the functional Q-body should be assembled on the yeast cell surface, and high-throughput screening of Q-bodies can be achieved using flow cytometry.

**Figure 1:**
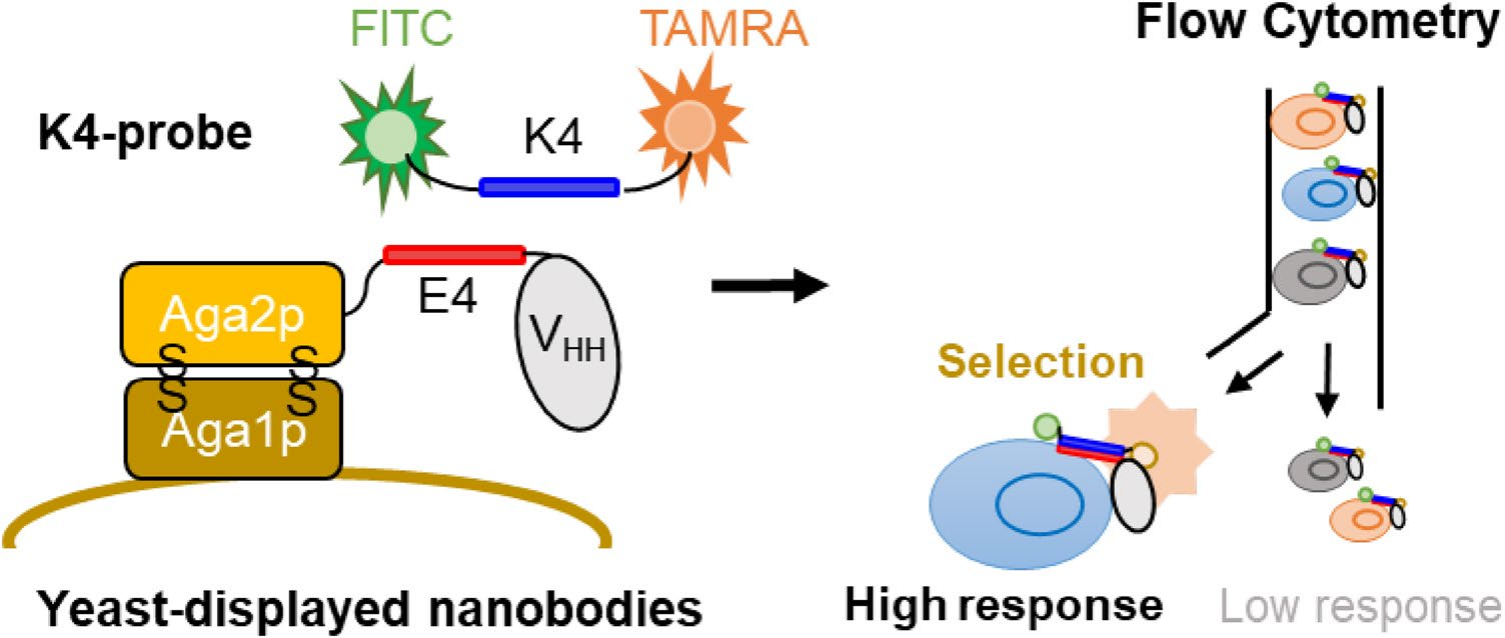
Schematic image of yeast-displayed mini Q-body and its selection steps. Nanobody-based Q-body (mini Q-body) was assembled on the yeast cell surface via E4/K4 interaction. To select potent mini Q-bodies, K4 peptide labeled with FITC and TAMRA was used to evaluate the quenching and de-quenching of TAMRA by flow cytometry.

In this study, a nanobody-based mini Q-body recognizing methotrexate (MTX) was assembled on the yeast cell surface and MTX was detected by flow cytometry. In addition, multiple nanobody candidates recognizing human serum albumin (HSA) were applied to this system to demonstrate that a highly responsive Q-body can be selected (Fig. 1).

## Results

### Assembly of Q-body on the yeast cell surface

First, a nanobody-based Q-body was chosen as a candidate for displaying the Q-body because of its low molecular weight and high stability, which could be beneficial in the cloud of the yeast cell surface. In addition, the MTX-recognizing nanobody was previously proven to function as a Q-body with sixfold response^11^, which suggested that MTX nanobody could be a suitable candidate for the proof-of-concept of the Q-body on the yeast cell surface. Also, an E4/K4 peptide pair was selected as a labeling tag because its high specificity and affinity will be suitable for assembling Q-bodies on the yeast cell surface, where various endogenous proteins coexist.

To this end, E4 peptides with or without C-terminal MTX nanobody were displayed along with the (GGGGS)_3_ linker and Aga2p at the N-terminal. The linker peptide between E4 peptide and the MTX nanobody was kept the same as previously described^22^. As shown in Supplementary Fig. S1, both E4 peptide and MTX nanobody with the N-terminal E4 peptide were successfully displayed on the yeast cell surface. In addition, the yeast-displayed MTX nanobody showed antigen-binding activity (*K*_D_: 22.4 nM) (Supplementary Fig. S2), which was in good agreement with that previously described^11^. This suggests that a sufficient amount of E4 peptide and MTX nanobody to evaluate the Q-body was displayed on the yeast cell surface without preventing antigen-binding activity.

**Figure 2:**
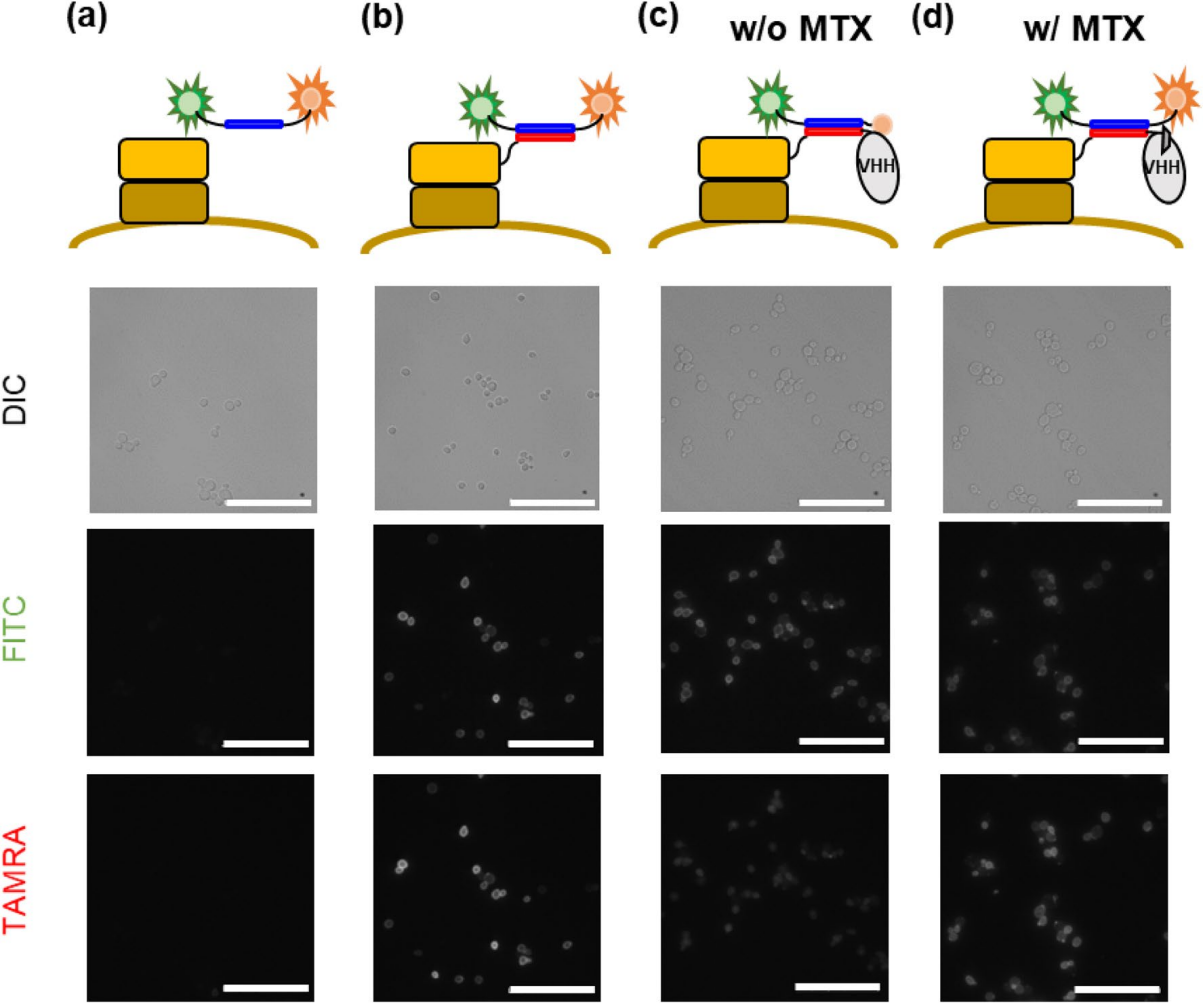
Differential interference contrast (DIC) (upper) and green/red fluorescence images (lower) of yeast cells displaying E4 or E4-MTX VHH labeled with FITC-K4-TAMRA. (**a**) Non-displayed yeast. (**b**) E4-displayed yeast. (**c**) E4-MTX VHH-displayed yeast without MTX. (**d**) E4-MTX VHH-displayed yeast with 1 µM MTX. Scale bars (white): 50 µm.

**Figure 3:**
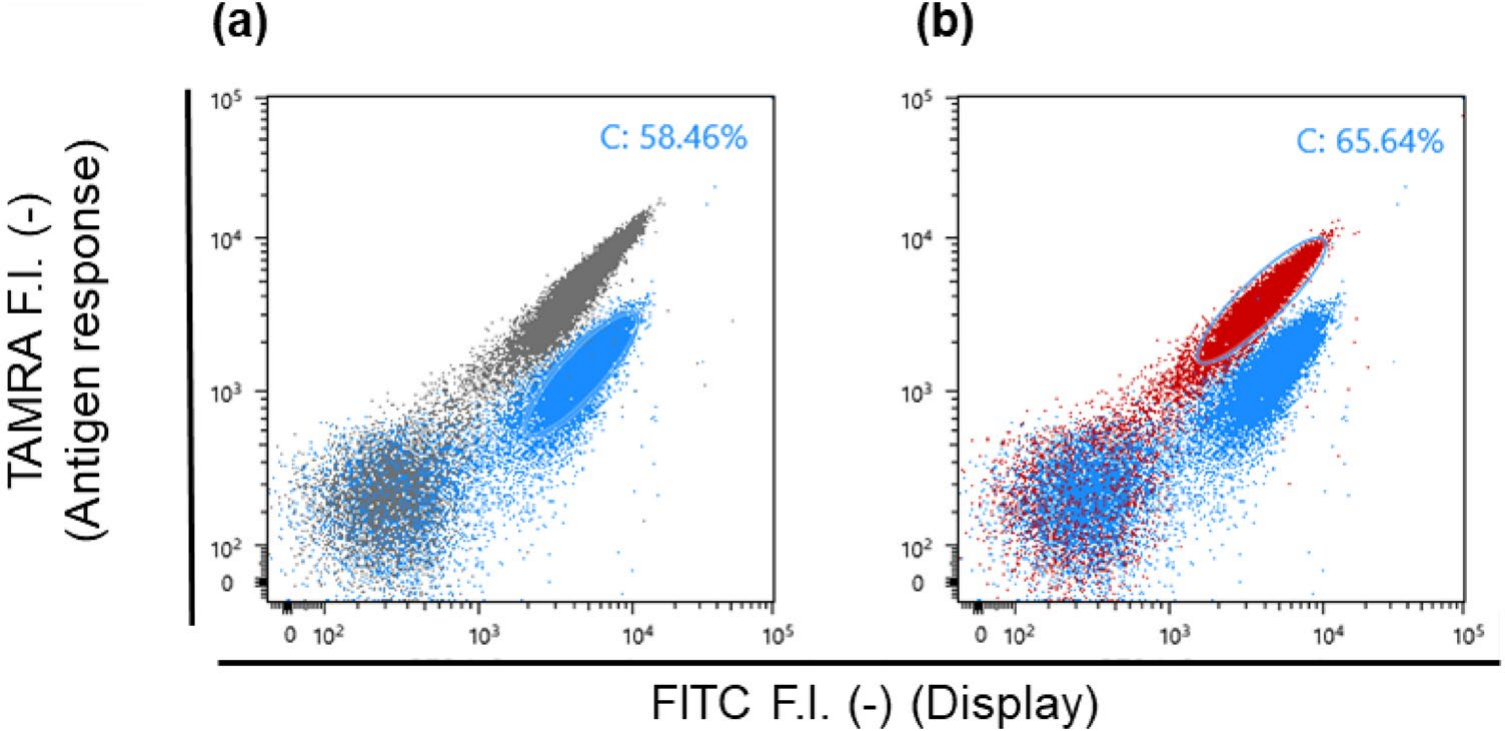
Flow cytometric analysis for yeast cells displaying E4-MTX VHH labeled with FITC-K4-TAMRA. (**a**) Analysis of quenching. (**b**) Analysis of de-quenching. Grey dots represent the yeast cell displaying E4 peptide. Blue dots represent the yeast cell displaying MTX VHH in the absence of MTX. Red dots represent the yeast cell displaying MTX VHH in the presence of 1 µM MTX.

Afterwards, K4 peptides labeled with fluorescein isothiocyanate (FITC) at the N-terminus and tetramethyl rhodamine (TAMRA) at the C-terminus was used to assemble the Q-body. The N-terminal FITC showed minimal fluorescence intensity change due to FRET efficiency change^22^, which made it easy to interpret the Quenching-based response of TAMRA. Also, the C-terminal TAMRA was attached to Cys through a C6 linker, which was enough in length to access the Trp residues^11,22^. Initially, two types of K4 probes with or without FITC at the N-terminus (FITC-K4-TAMRA and K4-TAMRA respectively) were constructed as previously described^22^, and the molecular weights (observed mass) of both peptides were confirmed to be almost the same as the previously obtained ones (data not shown). The yeast-displayed E4 peptide was successfully labeled with the synthesized FITC-K4-TAMRA, while the non-displaying yeast was not labeled (Fig. 2a, b, Supplementary Fig. S5), which indicated that the K4-probe was specifically labeled with the E4 peptide displayed on yeast cells, and that the yeast-displayed E4 peptide labeled with FITC-K4-TAMRA showed both FITC and TAMRA fluorescence at a mean of approximately 10^4^ (Fig. 3a; gray). Compared with the yeast labeled with K4-TAMRA or FITC-K4-C (Supplementary Fig. S3, Table S1), the fluorescence intensity of FITC decreased, while that of TAMRA increased, which suggested that Förster resonance energy transfer (FRET) from FITC to TAMRA occurred (The FRET efficiency of FITC-K4-TAMRA was calculated using the following formula, where $E$, $F_{{{\text{DA}}}}$, and $F_{{\text{D}}}$ represent FRET efficiency, fluorescence intensity of donor (FITC) in the presence of acceptor (TAMRA), and fluorescence intensity of donor in the absence of acceptor, respectively.

$$E = 1 - \frac{{F_{{{\text{DA}}}} }}{{F_{{\text{D}}} }}$$

By using the values in Supplementary Fig. S1, the FRET efficiency was calculated as 0.67). Consequently, TAMRA, which is not efficiently excited by a 488 nm laser, showed sufficient fluorescence to be observed by flow cytometry.

Therefore, the MTX nanobody-displaying yeast was also labeled with FITC-K4-TAMRA to assemble the Q-body on the yeast cell surface. The yeast-displayed MTX mini Q-body showed good quenching in the absence of MTX (Fig. 2c). Furthermore, it showed good dequenching in the presence of 1 µM MTX (Fig. 2d). Additionally, according to the means of TAMRA normalized by the mean of FITC in Fig. 3, the yeast-displayed MTX mini Q-body showed good quenching and a 3.28-fold fluorescence increase in the presence of MTX. It is worth noting that each dot plot population was discriminated, which suggested that the potent mini Q-body like the one recognizing MTX could be selected by fluorescence-activated cell sorting (FACS). In addition, when the dose–response analysis was performed using a yeast-displayed Q-body, it showed an ideal titration curve with a threefold response (Supplementary Fig. S6a, b), which indicates that the yeast-displayed min Q-body recognizing MTX could be used for the quantification of MTX in therapeutic drug monitoring.

### Selection of “mini Q-body” from anti-HSA nanobodies

**Figure 4:**
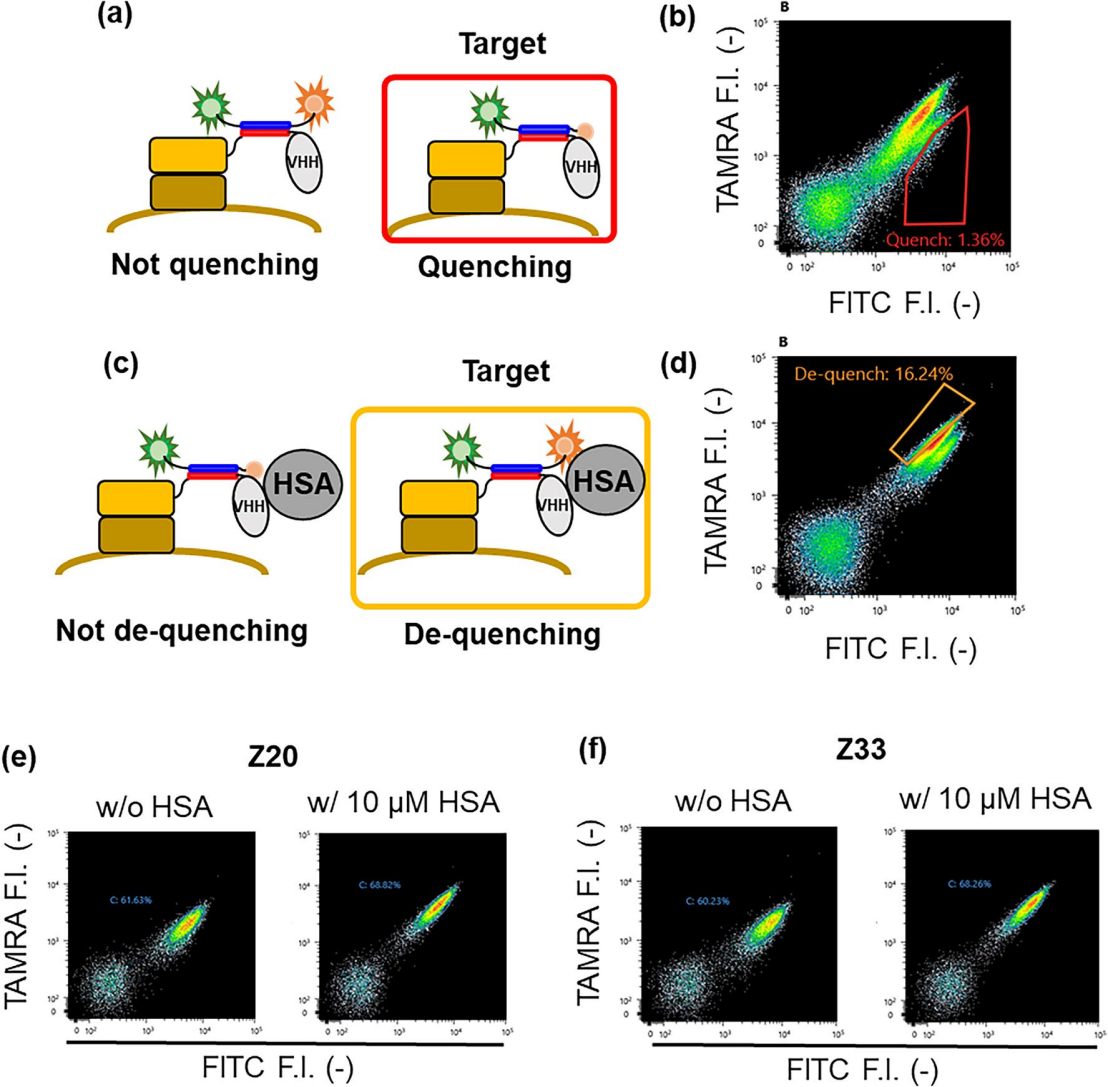
Selection of potent anti-HSA mini Q-body displayed on the yeast cell surface. (**a**, **c**) Schematic image of selection steps for quenching clones (**a**) and de-quenching clones (**c**). (**b**, **d**) Flow cytometric analysis in the selection of quenching clones (**b**) and the selection of de-quenching clones (**d**). Each clone was selected by the red gate or the yellow gate respectively. (**e**, **f**) Flow cytometric analysis of selected yeast displayed anti-HSA mini Q-bodies Z20 (**e**) and Z33 (**f**).

To demonstrate that the potent mini Q-body can be selected by using the above yeast surface display, 28 anti-HSA nanobodies, selected from a semi-synthetic alpaca V_HH_ phage display library^23^, were used as a model of antibody candidates for mini Q-bodies. Before starting, to confirm that the anti-HSA nanobodies can work on the yeast cell surface, five nanobodies, each of which has more than three tryptophans in different positions, were selected. Every nanobody was successfully displayed and showed antigen-binding activity (Supplementary Fig. S4). In addition, they were successfully labeled with FITC-K4-TAMRA, and one of them showed a quenching and medium fluorescence response (1.6-fold increase) (Supplementary Fig. S5, Table S2). Therefore, to obtain higher responsive clones, a plasmid mixture containing the other 23 nanobody genes was constructed using homologous recombination in yeast, and the nanobodies were displayed simultaneously. After labeling with FITC-K4-TAMRA, the potent yeast-displayed Q-bodies were selected by FACS in the following two steps: selection of clones that showed quenching in the absence of HSA (Fig. 4a) and selection of the clones that showed de-quenching in the presence of HSA (Fig. 4c).

Among the yeast-displayed Q-bodies, some showed deeper quenching, while others showed minimal quenching (Fig. 4b). In addition, among the well-quenched clones, some showed de-quenching, while others showed minimal de-quenching (Fig. 4d). After sequence analysis of the selected clones, only two nanobodies, Z20 and Z33, were selected. Both yeast-displayed Q-bodies showed an approximately 2.4-fold fluorescence increase as a single clone (Fig. 4e, f), and the yeast-displayed mini Q-body also showed an ideal titration curve (Supplementary Fig. S6c), indicating that the potent yeast-displayed Q-bodies were successfully selected by the yeast surface display. It is worth noting that Z20 and Z33 have the same amino acid sequence except for the N-terminal amino acid. In addition, nine of the 28 nanobodies including Z20, and Z33 show relatively high homology (> 90%) (Supplementary Fig. S7), and each showed a different fluorescent response (Supplementary Fig. S8, Table S3), which suggests that the response of the Q-body could be affected by only several amino acid differences. Nevertheless, unpredictable highly responsive clones such as Z20 or Z33 can be selected in this method, which demonstrates the usability of the combinatorial selection method for the successful high-throughput construction of Q-bodies.

### Construction of “mini Q-body” using *Escherichia coli* expressed parental nanobody

**Figure 5:**
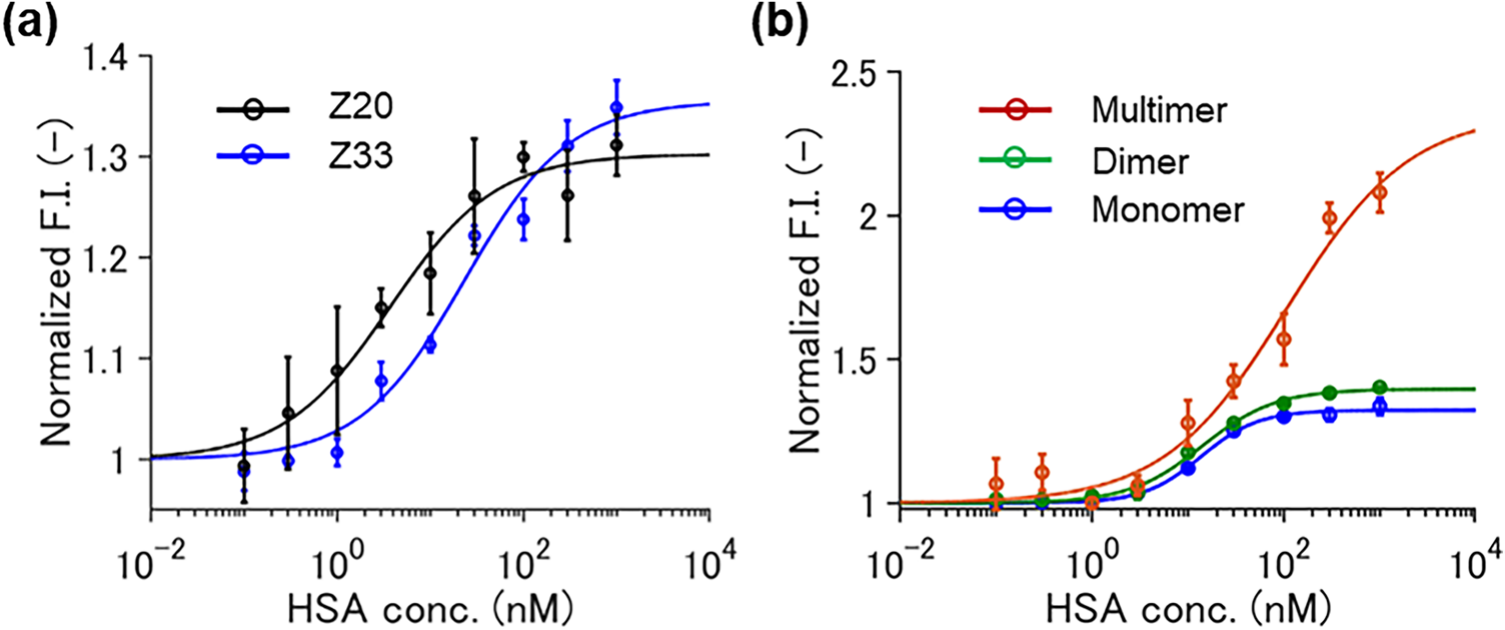
Dose–response curve of 5-TAMRA C6-labeled mini Q-body using selected nanobodies expressed by *E. coli* at 0–1 µM HSA. (**a**) Z20 and Z33 (**b**) Z33 at the state of one molecule (monomer), two proximate molecules (dimer) and multiple molecules captured on beads (multimer). Error bars represent ± 1 SD.

Finally, to confirm the validity of the selection method, mini Q-bodies were constructed using the selected nanobodies by *E. coli.* Since it was previously shown that MTX nanobody without linker labeled with 5-TAMRA C6 via Cys-tag showed the highest response^11^, the same construction method was adopted in this research. As shown in Supplementary Figs. S9–10, pSQ-Z20 and pSQ-Z33 were successfully labeled with 5-TAMRA C6 and showed an approximately 1.5-fold quenching and complete dequenching. In addition, the EC50 of 5-TAMRA C6-labeled Z20 and Z33 exhibited 3.7 nM and 23.4 nM respectively (Fig. 5a), which indicates that the nanobodies selected by yeast surface display also can work as Q-bodies even when expressed by *E. coli.*

However, the fluorescence response was relatively modest compared with that on the yeast cell surface (Fig. 4e, f), which suggests that the fluorescence response on the yeast cell surface was not identical to that expressed by *E. coli*. Therefore, we further focused on the most responsive nanobody, Z33, and aimed to explain the difference between these two conditions. To this end, the yeast-displayed Z33 was directly shaved from the yeast cell surface by reducing the disulfide bond between Aga1 and Aga2^24^. When using the same reduction procedure as that in the construction of the Q-body, the Z33 was successfully shaved from the yeast cell surface, and showed antigen-binding activity (Supplementary Fig. S11). Although the antigen-binding response was relatively low, this was due to the low concentration of Z33 on the yeast cell surface. Afterwards, the yeast-displayed Z33 mini Q-body was shaved, and the fluorescence response was compared with that on the yeast cell surface. As shown in Supplementary Fig. S11, the yeast-displayed Z33 mini Q-body showed a different response. The yeast-displayed Q-body showed a 2.4-fold response, and the shaved one showed a 1.24-fold response, which is in good agreement with that expressed by *E. coli.*

Given these results, we hypothesized that the difference in Q-body response was caused by the high surface concentration on the yeast cell surface rather than the yeast expression system itself. Therefore, to mimic the yeast surface display, 5-TAMRA C6 labeled Z33 expressed by *E. coli* was captured on anti-FLAG magnetic beads via the C-terminal FLAG tag or dimerized with anti-FLAG IgG. As shown in Fig. 5b, the two types of pSQ-Z33 showed higher fluorescence responses, and the assembled Q-body on anti-FLAG magnetic beads showed a twofold response. In addition, the dimerized pSQ-Z33 showed an additional absorbance peak at 525 nm (Supplementary Fig. S12), which suggests the H-dimer formation between two TAMRA dyes, as was also observed in double-dye-labeled Fab-type Q-body^25^ and in an scFv Q-body in the absence of antigen^15^. The deeper quenching due to H-dimer formation of the dyes in the neighboring mini Q-bodies could at least partly explain the higher responses on yeast cells and on the beads.

## Discussion

In the present study, we successfully constructed a novel combinatorial method for the construction of potent mini Q-bodies by combining yeast surface display and E4/K4 peptide pairs. First, the MTX mini Q-body, which was already known to function as a Q-body, was successfully assembled on the yeast cell surface and showed a good quenching and 3.3-fold fluorescence response in the presence of MTX. In addition, among the anti-HSA nanobody candidates, which were first utilized in this work, the responsive mini Q-body was successfully selected and showed a 2.4-fold fluorescence increase on the yeast cell surface. Even when expressed in *E. coli*, the selected anti-HSA nanobodies showed a 1.4-fold increase in fluorescence response with higher sensitivity compared with that of an scFv Q-body previously described^10^. Although the fluorescence response was lower than that on the yeast cell surface, dimerization by IgG and further assembly on magnetic beads could reduce the gap in the fluorescence response between them.

Notably, we found that there are two types of Q-bodies in terms of their working mechanisms. One of the Q-bodies was derived from intramolecular quenching and the other from intermolecular quenching, which could complicate the interpretation of selected clones. Nevertheless, by utilizing our combinatorial method, we can select both types of mini Q-bodies, which means that the sequence space of a potent Q-body could expand not only for quantification in the liquid phase but also in the solid phase, such as in an antibody-conjugated nanocavities based immunoassay^26^ or magnetic beads-based immunoassay^27^, which can improve the sensitivity or usability of Q-body assays. In addition, yeast-displayed mini Q-bodies derived from both anti-MTX nanobody and anti-HSA nanobody can be directly utilized to quantify the antigen without any purification steps. Such cell-based biosensors could meet the need for easy-to-use and low-cost testing, for example, in food safety and quality control^28^.

Compared with MTX nanobody or other highly responsive Q-bodies, the selected anti-HSA Q-bodies showed modest responses. This is probably because we used only 30 clones, which were selected by phage display but were not selected by the performance as a Q-body. Therefore, future work will focus on the construction of a synthetic library suitable for potent Q-bodies and directed evolution for various Q-bodies that do not achieve sufficient sensitivity in practical applications by combining recent novel evolutionary approaches using yeast surface display method^29^.

## Methods

### Materials

*Escherichia coli* XL-10 Gold (Agilent, Santa Clara, CA, USA) and SHuffle T7 Express lysY (New England Biolabs Japan, Tokyo, Japan) were used for general cloning and protein expression, respectively. EBY100 (ATCC**,** Manassas, VA, USA) was used for yeast cell surface display. Plasmid pYD1-mSA encoding N-terminal Aga2 protein and monomeric streptavidin was kindly given by Sheldon Park (Addgene #39865). The In-Fusion HD cloning kit, minimal SD base, -Trp/-Ura DO supplement, and TALON metal affinity resin were obtained from Takara-Bio (Shiga, Japan). Restriction enzymes were purchased from New England Biolabs (Tokyo, Japan). KOD-plus-neo and Ligation High Ver. 2 were from Toyobo (Osaka, Japan). The PureYield plasmid miniprep, Wizard SV Gel and PCR clean-up kits were purchased from Promega (Madison, WI, USA). The Frozen-Ez Yeast Transformation II kit was purchased from Zymo Research (Irvine, CA, USA), while the yeast nitrogen without amino acids and FITC-5-maleimide was obtained from Thermo Fisher Scientific. Anti-FLAG M2 magnetic beads, 3 × DYKDDDDK peptide, Biotin-PEG_2_-amine, and HSA were obtained from Sigma-Aldrich (St. Louis, MO, USA). 5-TAMRA-C6-mal cells were obtained from Setareh Biotech LLC (Eugene, OR, USA). MTX and zymolyase 100 T were obtained from Nacalai Tesque (Kyoto, Japan). PE anti-DYKDDDDK IgG and streptavidin-PE were purchased from Miltenyi Biotec. Unless otherwise indicated, all other chemicals and reagents were obtained from Sigma-Aldrich, Dojindo (Kumamoto, Japan), or Fujifilm-Wako Pure Chemicals (Osaka, Japan). The water used was purified using a Milli-Q water purification system (Merck Millipore, Burlington, MA, USA).

The following oligonucleotides (5′–3′) were synthesized by Eurofins Genomics (Tokyo, Japan):

- pYD1_back: AGTAACGTTTGTCAGTAATTGC.
- pYD1_for: GTCGATTTTGTTACATCTACAC.
- NheI_back: TCAGCTAGCATGGCTGAAATCGCTGC.
- BamHI_for: GTGGTGGTGGTGGTGGTGCTC.
- BspEI_back: CGTTCCGGACGCGTTCCTGAAACGC.
- Infusion_AgeI_back_Z1: GAAGGGAGGCACCGGTGAGGTGCAGCTCGTG.
- Infusion_AgeI_back_Z4: GAAGGGAGGCACCGGTCAGGTGCAGCTCGTG.
- Infusion_AgeI_back_Z9: GAAGGGAGGCACCGGTCAGTTGCAGCTCGTG.
- Infusion_AgeI_back_Z30: GAAGGGAGGCACCGGTGAGTTGCAGCTCGTG.
- Infusion_BamHI_for_Z: CCTTGTAGTCGGATCCTGCGGCCGCAGAGG.
- Inf_XhoI_Z_for: GGTGGTGGTGCTCGAGTGCGGCCGCAGAGGC.

The following peptides were synthesized at LifeTein (Hillsborough, NJ, USA):

- K4-C: NH_2_-KIAALKEKIAALKEKIAALKEKIAALKEC-COOH (M.W. 3137).
- FITC-K4-C: FITC-NH-KIAALKEKIAALKEKIAALKEKIAALKEC-COOH (M.W. 3639).

### Construction of yeast display plasmids

pYD1-E4 and pYD1-E4-MTXVHH were constructed to prove the concept of the yeast-displayed Q-body. Initially, two DNA fragments encoding the E4 peptide and the other encoding MTX-V_HH_ with N-terminal E4 peptide were amplified from pE4-MTXVHH as a template using primers NheI_for and BamHI_back, or NheI_for and T7 terminator, respectively. After the DNA fragments were digested using *Nhe*I and *Bam*HI, both were cloned into the *Nhe*I- and *Bam*HI-digested vector of pYD1-mSA using Ligation High ver. 2.

Twenty-eight pYD1-HSAVHH clones encoding different V_HH_ sequences were constructed using two methods. Five plasmids encoding Z02, Z06, Z08, Z18, and Z19 were constructed using In-Fusion cloning, and the other 23 plasmids were constructed simultaneously using homologous recombination in the yeast cell. Initially, pYD1-E4-BGPscFv, in which the restriction site of *Age*I on the GAL1 promoter was removed using BspEI_back, was constructed. Then, 28 HSAV_HH_ genes were amplified using Infusion_AgeI_back_Zn (n = 1, 4, 9, 30) and Infusion_BamHI_for, respectively. The DNA fragments encoding Z02, Z06, Z08, Z18, and Z19 were cloned into *Age*I- and *Bam*HI-digested pYD1-E4-BGPscFv by In-Fusion cloning, and the DNA fragments encoding the other 23 HSA V_HH_ genes were mixed with *Age*I- and *Bam*HI-digested pYD1-BGPscFv at a molar ratio of 5:1, followed by homologous recombination in the cell.

### Nanobody display on the yeast cell surface

The constructed plasmid or vector/insert mixture was transformed into EBY100 using the Frozen-Ez Yeast Transformation II kit and cultured at 30 °C for 2–3 days in a culture plate containing minimal synthetic defined base (including a yeast nitrogen base, ammonium sulfate, glucose), -Trp/-Ura DO supplement [SD (-W/-U)], and 2% agar. A single colony was picked and grown at 30 °C in 3 mL of SD (-W/-U) medium overnight, and ~ 1 mL of this culture was used to inoculate 20 mL of SD (-W/-U) medium at 0.2–0.3 OD_600_, followed by culturing at 30 °C. After the OD_600_ reached 0.4–0.6, the medium was exchanged with SG (-W/-U) medium, in which a glucose of SD (-W/-U) medium was replaced with a galactose, and the cells were incubated for an additional 18–24 h at 20 °C to display the proteins encoded on the plasmid.

### Preparation of K4-probe

One of the coiled-coil forming peptides, K4, was labeled with TAMRA via a maleimide-thiol reaction, as previously described^24^. Briefly, K4 peptide with C-terminal cysteine (10 nmol) and 5-TAMRA C6 maleimide (11 nmol) were dissolved in 50 µL of Milli-Q water and vigorously mixed for 2 h at 25 °C. The labeled peptide (FITC-K4-TAMRA) was purified using RP-HPLC (Chromaster, Hitachi High-tech, Tokyo, Japan) and identified by MALDI-TOF-MS (UltrafleXtreme, Bruker, Billerica, MA, USA).

### Preparation of yeast-displayed Q-body

After inducing the display, the yeast cells (~ 5 × 10^6^ cells) were collected by centrifugation at 14,000 × g for 1 min at 4 °C, and the pellet was washed with 1 mL phosphate-buffered saline (PBS) containing 5% immunoblock (DS Pharma Biomedical, Osaka, Japan) (PBS-B). After the pellet was resuspended in 100 µL PBS-B, FITC-K4-TAMRA was added at a final concentration of 100 nM, followed by incubation for 15 min at 4 °C, followed by two washings with 1 mL PBS-B.

### Microscopic observation

After the yeast displayed Q-body was resuspended in 50 µL PBS-B, microscopy was performed with an IX 71 inverted microscope (Olympus, Tokyo, Japan). Samples were observed under a 60 × objective lens. Fluorescein was excited at 460–480 nm and emission at 495–540 nm was observed. TAMRA was excited at 545–580 nm and emission at > 610 nm was observed.

### Analysis and selection of yeast-displayed Q-body by FACS

After the yeast-displayed Q-body was resuspended in 500 µL of PBS-B, flow cytometric analysis was performed using an SH-800 cell sorter (Sony, Tokyo, Japan). Three thousand events were measured in each analysis. A blue laser (488 nm) and two detection filters (525/50 and 585/30) were used to measure the fluorescence intensities of FITC and TAMRA, respectively. The obtained data were analyzed using the control software, and the means of FITC and TAMRA were calculated to check the quenching in the absence of the antigen, and the fluorescence response in the presence of the antigen. About 5000 cells in the gate that showed quenching or de-quenching were sorted and used for the next round of analysis.

The yeast cells selected by FACS were cultured in 2 mL SD (-W, -U) medium for 2 days, and the nanobody display and selection procedure were continued. Finally, 1-day culture medium was subsequently cultivated on SD (-W, -U) agar plates for 2 days. A single colony was picked, and a nanobody display was performed.

### Subcloning of selected nanobody and expression in *E. coli*

pSQ-Z20 and Z33 were constructed to express Z20 and Z33, respectively, in *E. coli*. Initially, two fragments, one encoding Z20 and the other encoding Z33, were amplified from pYD1-Z20 and pYD1-Z33 in zymolyase-treated yeast cells as a template using pYD1-back and Inf_XhoI_for, respectively. The DNA fragments were cloned into the *Age*I- and *Xho*I-digested vectors of pSQ-MTXVHH using Ligation High ver.2. The constructed plasmid was transformed into SHuffle T7 Express lysY cells, and the nanobodies were expressed and purified as previously described^11^.

### Q-body preparation and dose-dependency measurement

The purified Z20 and Z33 were labeled with maleimide dye as previously described^11^, and the fluorescence intensity was measured using a fluorescence plate reader (Clariostar, BMG Labtech). Initially, each mini Q-body was diluted to 5 nM in PBS supplemented with 0.05% Tween 20 (PBST) or 7 M guanidium hydrochloride (GdmHCl) containing 100 mM dithiothreitol (DTT) in PBST to denature the protein and measure the degree of quenching. In addition, HSA was added to the solution at nine concentrations (0.1, 0.3, 1, 3, 10, 30, 100, 300, and 1000 nM) to confirm the dose-dependency. Each solution (80 μL) was applied to a 96-well black microplate and fluorescence was measured at 535 nm excitation and 585 nm emission wavelengths. Dose–response curves were drawn by fitting the intensities at the maximum emission wavelength using the curve fitting function of MATLAB (Mathworks, Natick, MA). The EC50 value was calculated from the curve fitting to a modified 4-parameter logistic (4PL) equation, as previously described^11^.

In addition, 5-TAMRA C6 labeled Z33 was dimerized using anti-FLAG IgG or captured on anti-FLAG magnetic beads. For these tests, 5-TAMRA C6 labeled Z33 was purified using a His spin trap column instead of anti-FLAG M2 magnetic beads. The purified Z33 was reacted with anti-FLAG M2 IgG at a ratio of 2:1 at 25 °C for 30 min to be dimerized via an anti-FLAG M2 IgG molecule. To mimic the yeast surface display, 5-TAMRA C6 labeled Z33 was kept on anti-FLAG M2 magnetic beads without elution during the purification steps, in which the 5-TAMRA C6 labeled Z33 was captured on the bead surface via anti-FLAG M2 IgGs. The dose-dependencies for the both samples were confirmed according to the aforementioned procedure.

### Fluorescence analysis of yeast-displayed mini Q-body

The dose–response of the yeast-displayed mini Q-body was measured using either a fluorescence spectrophotometer Model FP-8500 (Jasco, Tokyo, Japan) or a fluorescence microplate reader Clariostar (BMG Labtech Japan, Saitama, Japan). After the yeast-displayed mini Q-body was assembled as described previously, it was diluted to 0.3 OD_600_, and the antigen (MTX or HSA) was added to the solution at eight concentrations (1, 3, 10, 30, 100, 300, 1000, and 3000 nM). For fluorescence spectral measurements, each solution (250 μL) was added in a 5 mm × 5 mm quartz cell (Starna Scientific, Hainault, UK), and the fluorescence spectrum was measured at 485 nm excitation and 510–650 nm emission wavelengths. The measurement temperature, excitation/emission bandwidths, and scanning speed were set to 25 °C, 5 nm, 200 nm/min, respectively. For fluorescence ratiometric measurement, each solution (80 μL) was applied to a 96-well black microplate and fluorescence was measured at 483 nm excitation and 535 nm or 585 nm emission wavelengths. Dose–response curves were drawn by fitting the fluorescence intensity of each concentration at 585 nm normalized to that at 535 nm (R/G ratio) using a curve fitting function as described.

### Shaving and Analysis of yeast-displayed mini Q-body

The shaving of the yeast-displayed mini Q-body was performed using a mild reduction treatment. Initially, the yeast-displayed Z33 (~ 0.5 × 10^7^ cells) was reduced using tris(2-carboxyethyl) phosphine hydrochloride (TCEP-HCl) at 2.5 mM in 100 µL PBST at 30 °C for 20 min. The unreacted TCEP was oxidized by 10 mM 4-azidobenzoic acid (ABA)^30^, and the reduced mini Q-body was collected by centrifugation. The pellet (yeast cell) was labeled with PE-conjugated anti-FLAG IgG to confirm the degree of shaving. The antigen-binding activity of the supernatant (reduced Z33) was confirmed by ELISA. Briefly, HSA was immobilized in the wells of a transparent polystyrene microplate (Costar 3590, Corning-Costar Japan, Tokyo, Japan) at 4 °C overnight, blocked with 20% ImmunoBlock (DS Pharma Biomedical, Osaka, Japan) in PBS at 25 °C for 2 h, and washed three times with PBST. The twice-diluted supernatant in 80 μL of PBST containing 5% ImmunoBlock was applied and incubated for 1 h at 25 °C, followed by three washes with PBST. The bound nanobodies were probed with 100 μL of 1/10,000 diluted horseradish peroxidase-conjugated anti-FLAG antibody in PBST containing 5% ImmunoBlock for 30 min at 25 °C. After washing three times with PBST, 100 μL of substrate solution (200 μg/mL 3,3′,5,5′-tetramethylbenzidine and 0.3 μL/mL (v/v) hydrogen peroxide in 100 mM sodium acetate, pH 6.0) was applied to the well. After incubation for 2–3 min, the reaction was stopped with 50 μL per well of 1 M sulfuric acid, and the absorbance was determined at 450 nm with a reference at 655 nm using a microplate reader (SH-1000Lab, Corona Electric, Ibaraki, Japan). The yeast-displayed E4-peptide and Z33 (~ 0.5 × 10^7^ cells) labeled with FITC-K4-TAMRA were shaved using the aforementioned procedure, and fluorescence intensity in the presence or absence of 10 µM HSA was confirmed using a fluorescence plate reader in the same manner as with the 5-TAMRA C6 labeled mini Q-body.

## Supplementary Information

Supplementary Information.

## Abbreviations

ABA: 4-Azidobenzoic acid
Aga: a-Agglutinin
BGP-C7: C-terminal 7 residue peptide of bone Gla protein
BRET: Bioluminescence resonance energy transfer
CDR: Complementarity determining region
DTT: Dithiothreitol
Glu; E: Glutamic acid
ELISA: Enzyme-linked immunosorbent assay
Fab: Antigen-binding fragment of antibody
FACS: Fluorescence-activated cell sorting
FITC: Fluorescein isothiocyanate
Fv; V_H_ + V_L_: Antibody variable region
FRET: Förster (fluorescence) resonance energy transfer
Gly; G: Glycine
GdnHCl: Guanidine hydrochloride
HRP: Horse radish peroxidase
HSA: Human serum albumin
Lys; K: Lysine
*K*_d_: Dissociation constant
LOD: Limit of detection
MALDI-TOF-MS: Matrix assisted laser desorption/ionization-time of flight mass spectrometry
MTX: Methotrexate
PBS: Phosphate buffered saline
PBS-B: PBS containing 5% immunoblock
PBST: PBS containing 0.05% Tween 20
PE: Phycoerythrin
PET: Photoinduced electron transfer
POCT: Point-of-care testing
POI: Protein of interest
Q-body: Quenchbody
RP-HPLC: Reverse-phase high performance liquid chromatography
Ser; S: Serine
scFv: Single-chain variable region
SuCESsFul: Scaffold conjugated to environment-sensitive fluorophore
TAMRA: Tetramethyl rhodamine
TCEP-HCl: Tris(2-carboxyethyl) phosphine hydrochloride
TDM: Therapeutic drug monitoring
TMBz: 3,3′, 5,5′-Tetramethylbenzidine
U: Uracil
UQ-body: Ultra Q-body
V_H_: Variable region of antibody heavy chain
V_HH_: Camelid heavy-chain antibody variable domain
V_L_: Variable region of antibody light chain
Trp; W: Tryptophan
