# Supplementary material for: Evaluation and selection of potent fluorescent immunosensors by combining fluorescent peptide and nanobodies displayed on yeast surface
Source: Sci Rep. 2021 Nov 19;11:22590. doi: 10.1038/s41598-021-02022-7 (PMC8604967; doi:10.1038/s41598-021-02022-7)
Supplement: Supplementary file 1 — Supplementary Information. [file 41598_2021_2022_MOESM1_ESM.pdf]

Supporting Information for

Evaluation and selection of potent fluorescent immunosensors by combining fluorescent peptide and nanobodies displayed on yeast surface

Akihito Inoue[1], Takanobu Yasuda[1], Bo Zhu [2], Tetsuya Kitaguchi[2], Akikazu Murakami[3,4], Hiroshi Ueda[2*]

[1] *Graduate School of Life Science and Technology, Tokyo Institute of Technology, 4259-R1-18 Nagatsuta-cho, Midori-ku, Yokohama, Kanagawa 226-8503, Japan*

[2] *Laboratory for Chemistry and Life Science, Institute of Innovative Research, Tokyo Institute of Technology, Nagatsuta-cho, Yokohama, Kanagawa 226-8503, Japan*

[3] *Department of Oral Microbiology, Institute of Biomedical Sciences, Tokushima University Graduate School, 3-18-15 Kuramoto, Tokushima 770-8504, Japan*

[4] *RePHAGEN Co., Ltd. Uruma, Okinawa, 904-2234, Japan*

Table of Contents

Table S1. FRET analysis of K4 probes on the E4-displaying yeast by FACS. S-2
Table S2. Q-body response of five anti-HSA nanobodies. S-3
Table S3. Q-body response of homological anti-HSA nanobodies. S-3

Figure S1. Display level of E4 peptide with or without C-terminal MTX-V_{HH}. S-4
Figure S2. Antigen binding activity of yeast-displayed E4-MTXV_{HH}. S-5
Figure S3. Flow cytometric analysis of labeled K4-probe. S-6
Figure S4. Flow cytometric analysis of yeast-displayed anti-HSA nanobodies. S-6
Figure S5. Flow cytometric analysis of yeast-displayed anti-HSA mini Q-body. S-7
Figure S6. Dose responses of yeast-displayed mini Q-body. S-8

Figure S7. Sequence alignment of anti-HSA nanobodies. S-9
Figure S8. Flow cytometric analysis of homological anti-HSA mini Q-body. S-10
Figure S9. Fluorescent and CBB-stained images of anti-HSA mini Q-body. S-11
Figure S10. Fluorescence response analysis of anti-HSA mini Q-body. S-11
Figure S11. Shaving of anti-HSA nanobody (Z33) displayed on the yeast cell surface. S-12
Figure S12. UV spectra of dimerized 5-TAMRA C6-labeled Z33. S-13

Table S1. FRET analysis of K4 probes on the E4-displaying yeast by FACS.

Probe Name	Mean (FITC)	Mean (TAMRA)
FITC-K4-TAMRA	6816	7144
FITC-K4-C	20680	-
K4-TAMRA	-	1111

Table S2. Mini Q-body response of five anti-HSA nanobodies displayed on yeast surface.

Clone	Mean (TAMRA(–)) / Mean (Flu(–))		Response (+ HSA / – HSA) (–)
	- HSA	+ HSA	
E4	1.08	–	–
Z02	0.96	0.98	1.02
Z06	0.87	0.91	1.05
Z08	0.95	0.94	0.99
Z18	0.51	0.79	1.55
Z19	0.94	1.00	1.06

Table S3. Mini Q-body response of similar anti-HSA nanobodies displayed on yeast surface.

Clone	Mean (TAMRA(–)) / Mean (Flu(–))		Response (+ HSA / – HSA) (–)
	- HSA	+ HSA	
E4	1.08	–	–
Z07	0.90	0.83	0.93
Z16	0.30	0.32	1.05
Z18	0.50	0.74	1.48
Z20	0.37	0.75	2.06
Z22	0.46	0.81	1.75
Z23	0.39	0.68	1.73
Z26	0.40	0.46	1.14
Z30	0.79	0.77	0.97
Z31	0.42	0.46	1.11
Z33	0.32	0.78	2.42

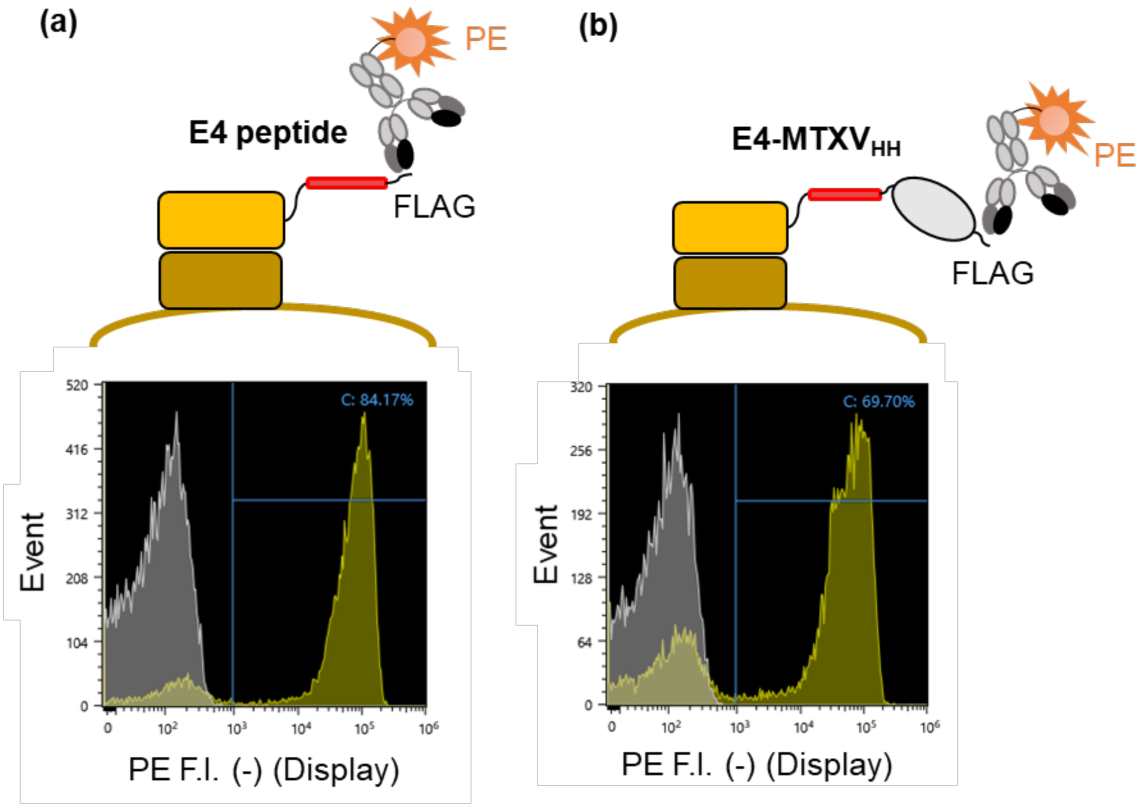

Figure S1. Display level of E4 peptide with or without C-terminal MTX-V$_{HH}$. The E4 peptide or E4-MTXV$_{HH}$ was orthogonally labelled with PE-conjugated anti-FLAG IgG (**Miltenyi Biotec**). The two histograms represent **(a)** E4 peptide and **(b)** E4-MTXV$_{HH}$, respectively (**yellow**), and compared to labelled yeast cells that do not display (**gray**). The fluorescence intensity of each cell was monitored at 585/30 nm upon excitation with a 488 nm laser.

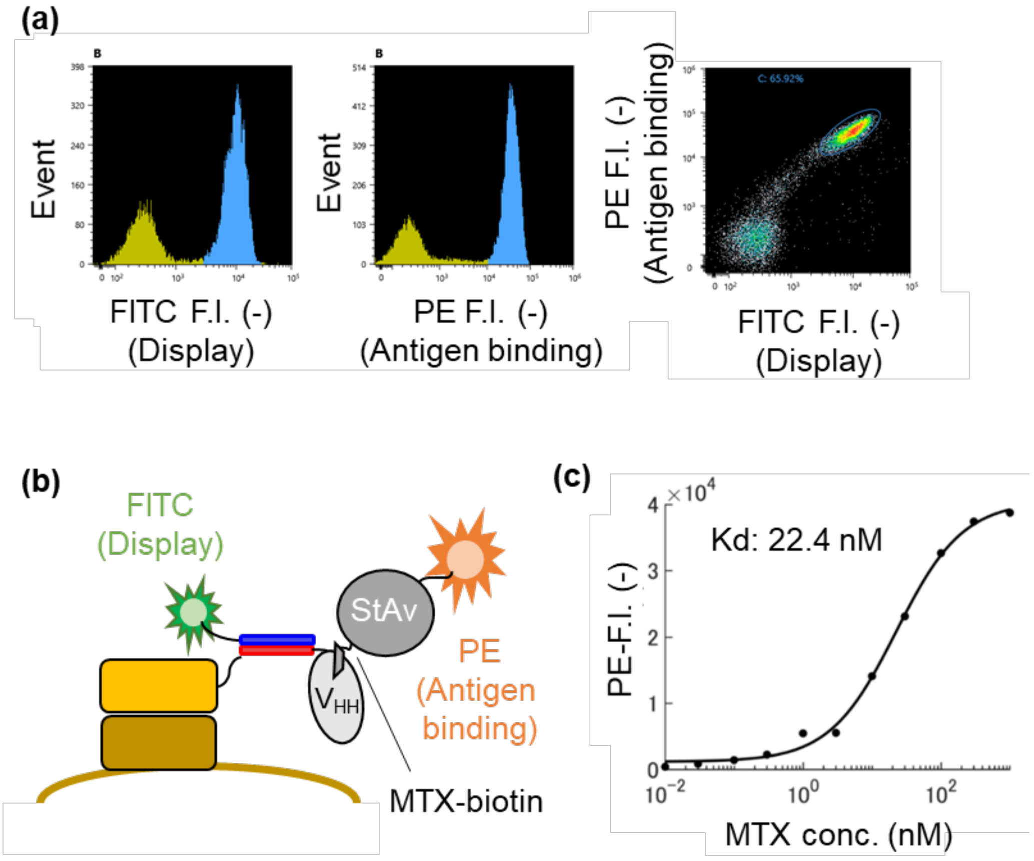

Figure S2. Antigen binding activity of yeast-displayed E4-MTXV$_{HH}$. Firstly, the E4-MTXV$_{HH}$ was orthogonally labelled with FITC-K4-C (LifeTein) and each concentration of biotinylated MTX, followed by mixed with PE-conjugated streptavidin (Miltenyi Biotec). **(a)** Histograms and a dot plot of FITC and PE when labeling with 1 μM biotinylated MTX. **(b)** Schematic image of fluorescence labeling **(c)** The sigmoid curve at 0~1 μM biotinylated MTX. The fluorescent intensity of PE was obtained from the dot plots at each concentration, and calculated the mean value using the internal software. The sigmoid curve was fitted using MATLAB software.

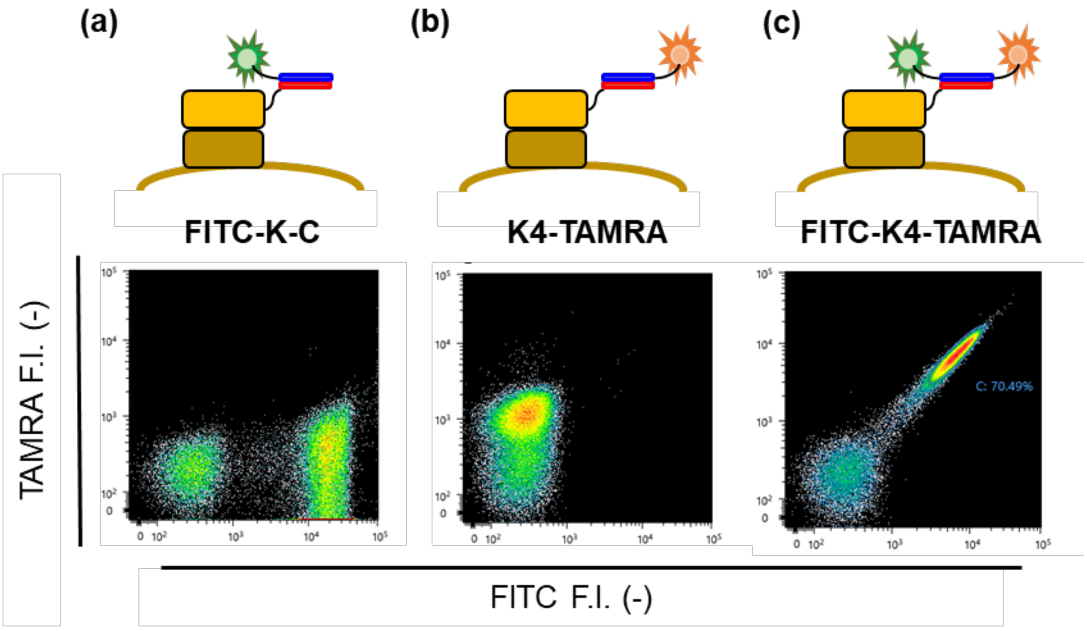

Figure S3. Flow cytometric analysis of labeled K4-probes. The yeasts displaying E4 peptides were labeled with **(a)** FITC-K4-C, **(b)** K4-TAMRA and **(c)** FITC-K4-TAMRA. The mean FITC and TAMRA fluorescence intensities are shown in **Table S1**.

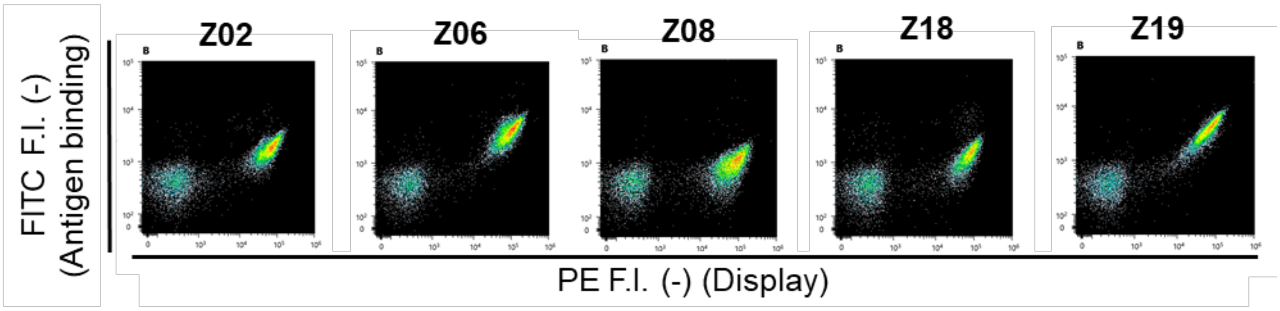

Figure S4. Flow cytometric analysis of the display level and the antigen-binding activity of anti-HSA nanobodies. Five anti-HSA nanobodies called Z02, Z06, Z08, Z18, and Z19 were orthogonally labeled with PE-conjugated anti-Flag antibody (Miltenyi Biotec) and 500 nM of FITC-labeled HSA. FITC and PE represent the antigen-binding activity and the display level, respectively.

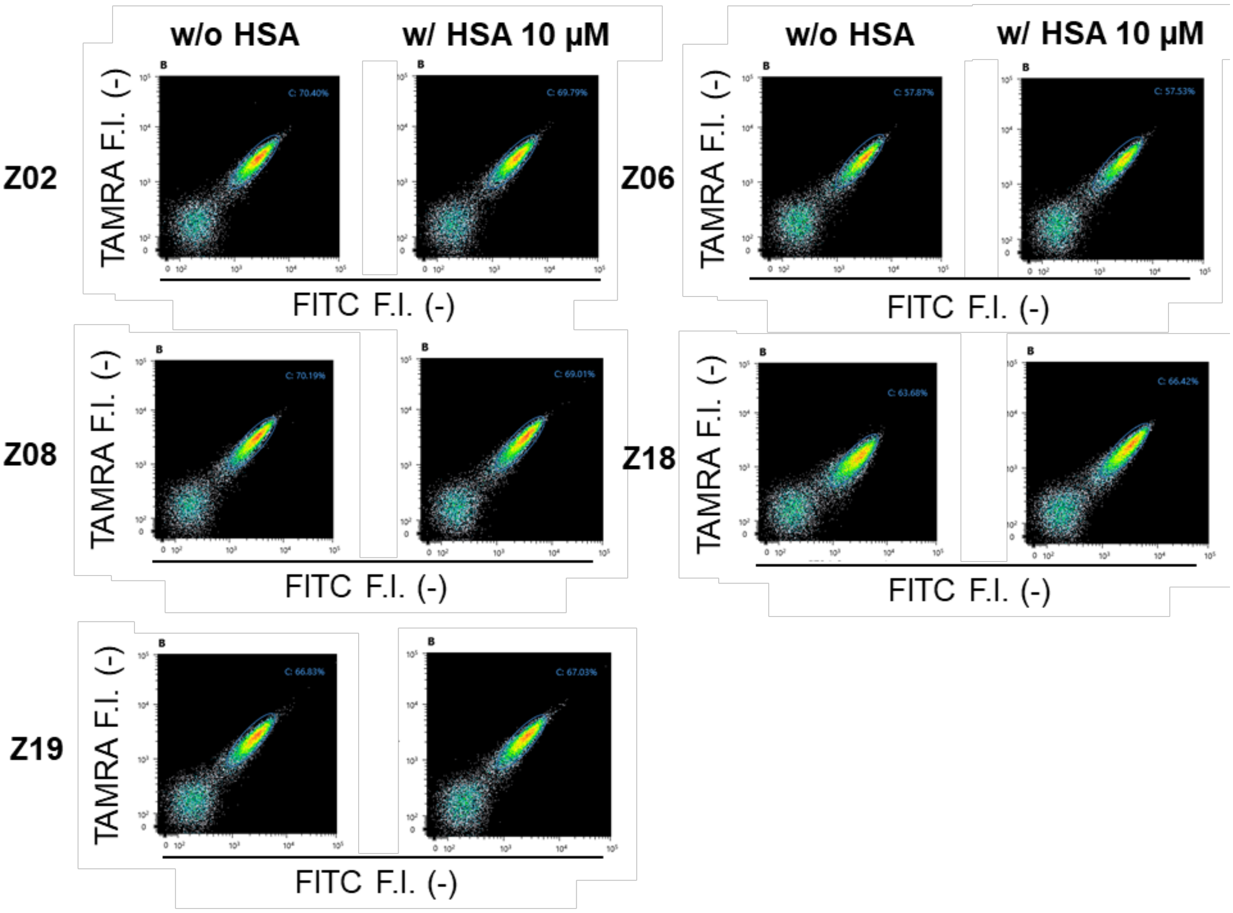

Figure S5. **Flow cytometric analysis of Q-body response for anti-HSA nanobodies**. Each yeast-displayed anti-HSA nanobody was labeled with FITC-K4-TAMRA, and the fluorescence response was calculated from the mean value of TAMRA divided by the mean of FITC in **Table S2**.

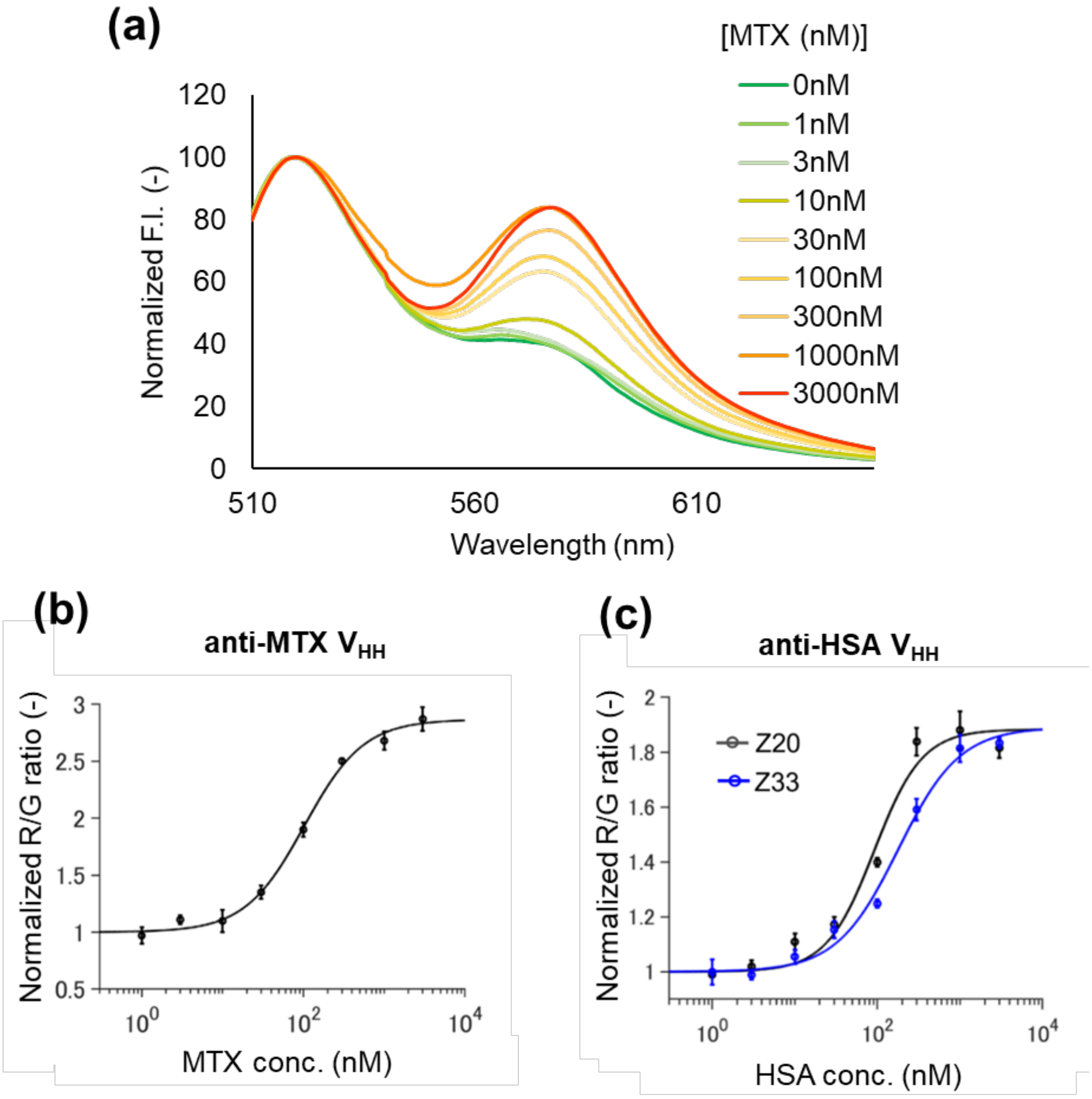

Figure S6. Dose-responses of yeast-displayed mini Q-body. (a) Fluorescence spectra of anti-MTX mini Q-body displayed on yeast cell surface upon adding MTX at indicated concentrations, which are normalized at emission maxima. **(b, c)** Dose-response curves of anti-MTX mini Q-body (b) and anti-HSA mini Q-body (c) displayed on the yeast cell surface. The yeasts were diluted to 0.3 OD_{600}. Error bars represent ±1 standard deviation (SD) (n=3)

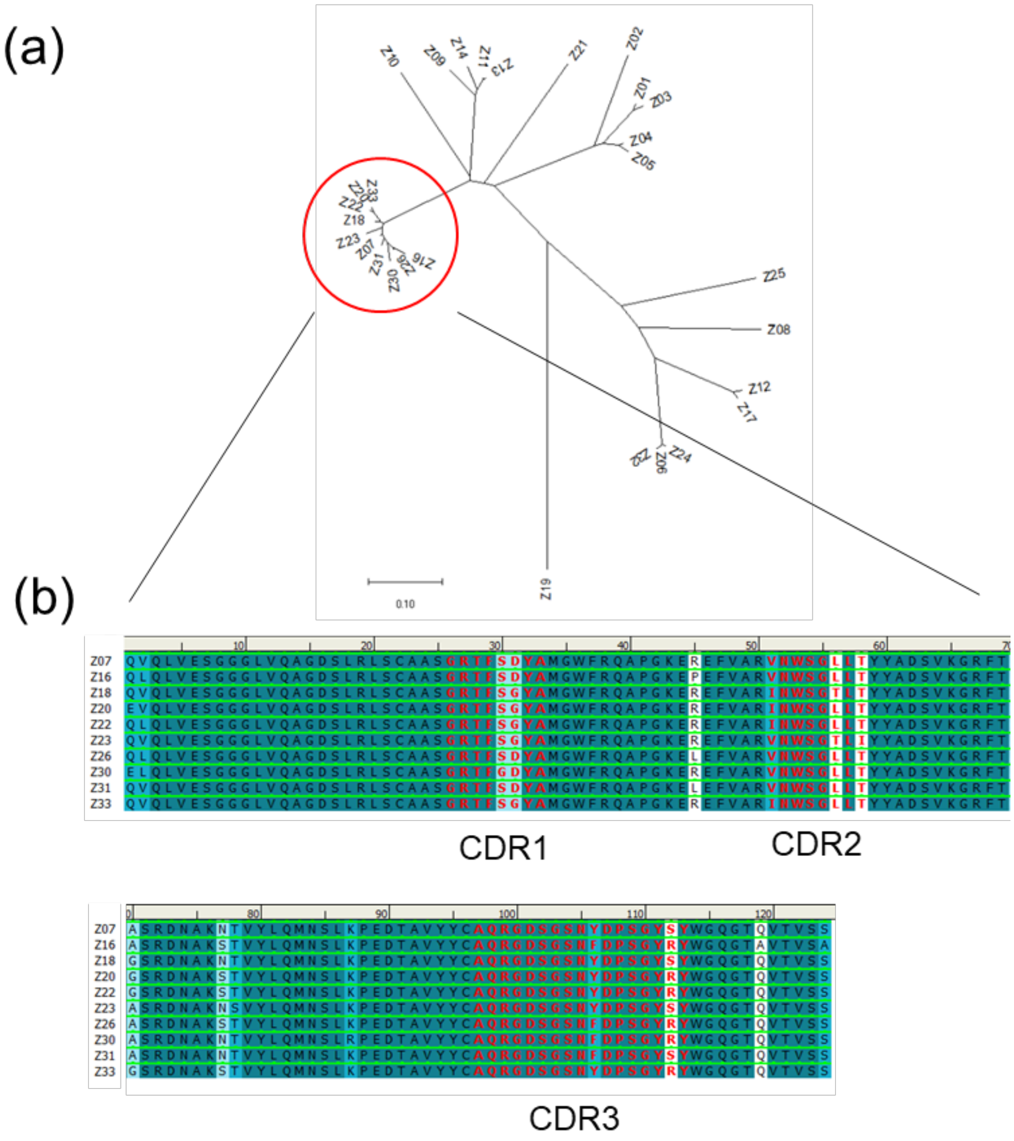

Figure S7. **Sequence alignment of anti-HSA nanobodies. (a)** Phylogenetic analysis of all anti-HSA nanobodies using MEGA-X (https://www.megasoftware.net). **(b)** Sequence alignment of anti-HSA nanobodies that show high homology (red circle in (a)). The amino acid residues with dark green background represent the same residues among eight anti-HSA nanobodies, while those with light green or white background represent different ones among them. The residues depicted in red are in CDR detected by IMGT numbering using Discovery Studio Client, 2018 (Dassault Systèmes BIOVIA, Vélizy-Villacoublay, France).

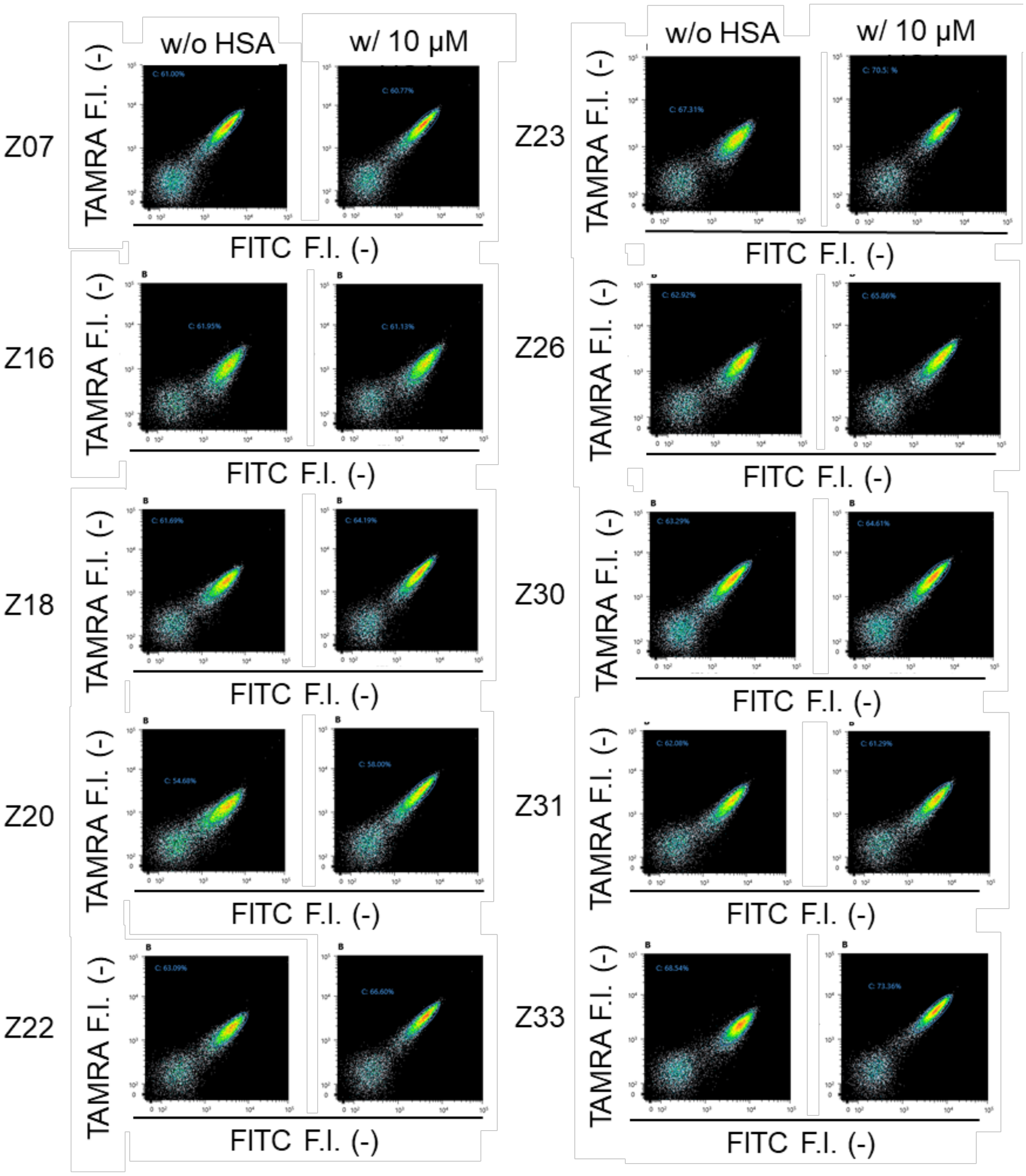

Figure S8. **Flow cytometric analysis of Q-body response for homological anti-HSA nanobodies in Fig S9**. The fluorescence response of each yeast-displayed mini Q-body was calculated and shown in **Table S3**.

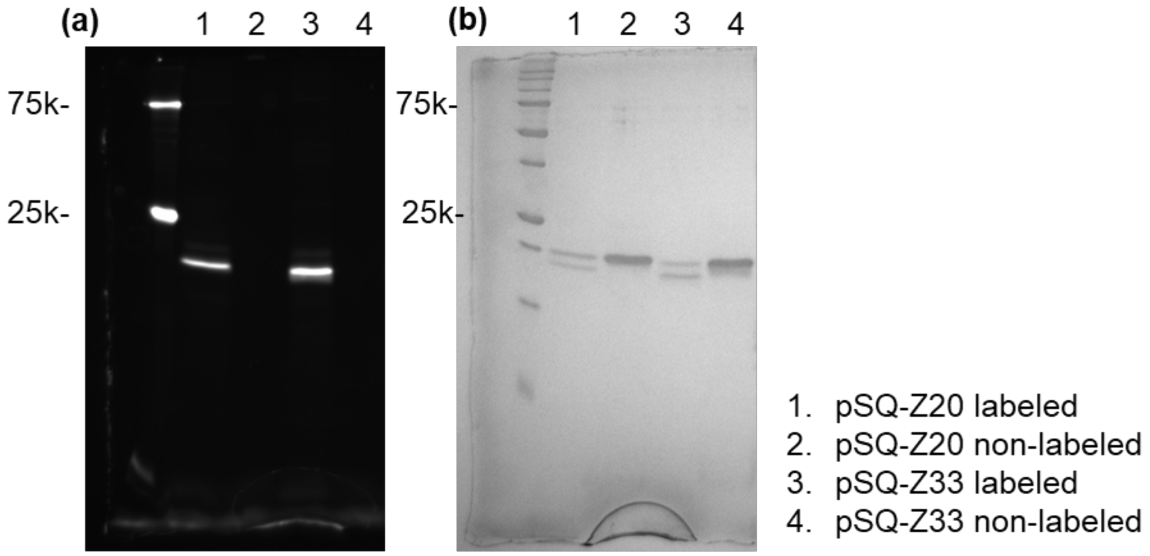

Figure S9. Fluorescent and CBB-stained images of 5-TAMRA C6-labeled nanobodies. **(a)** Fluorescent and **(b)** CBB-stained images of SDS-PAGE for the fluorescence-labeled nanobodies (HSA nanobodies, Z20 and Z33). The same gel is presented in both (a) and (b) without cropping.

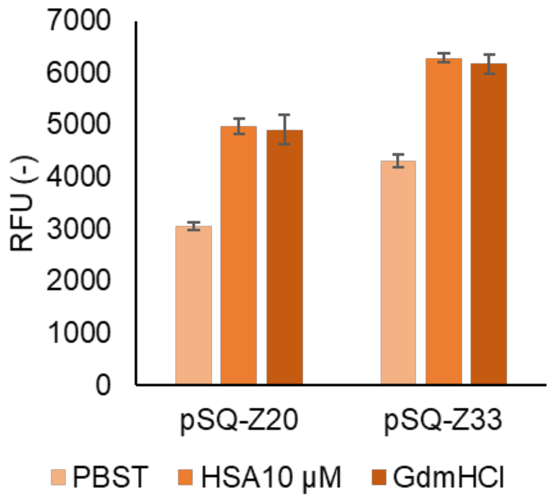

Figure S10. Quenching and antigen-dependent recovery of 5-TAMRA C6 labeled nanobodies. Thin red represents the fluorescence intensity of Q-body in PBST at 1 nM, while medium and dark red represent that in denaturant and 10 μM HSA, respectively. Error bars represent ±1 standard deviation (SD) (n =3)

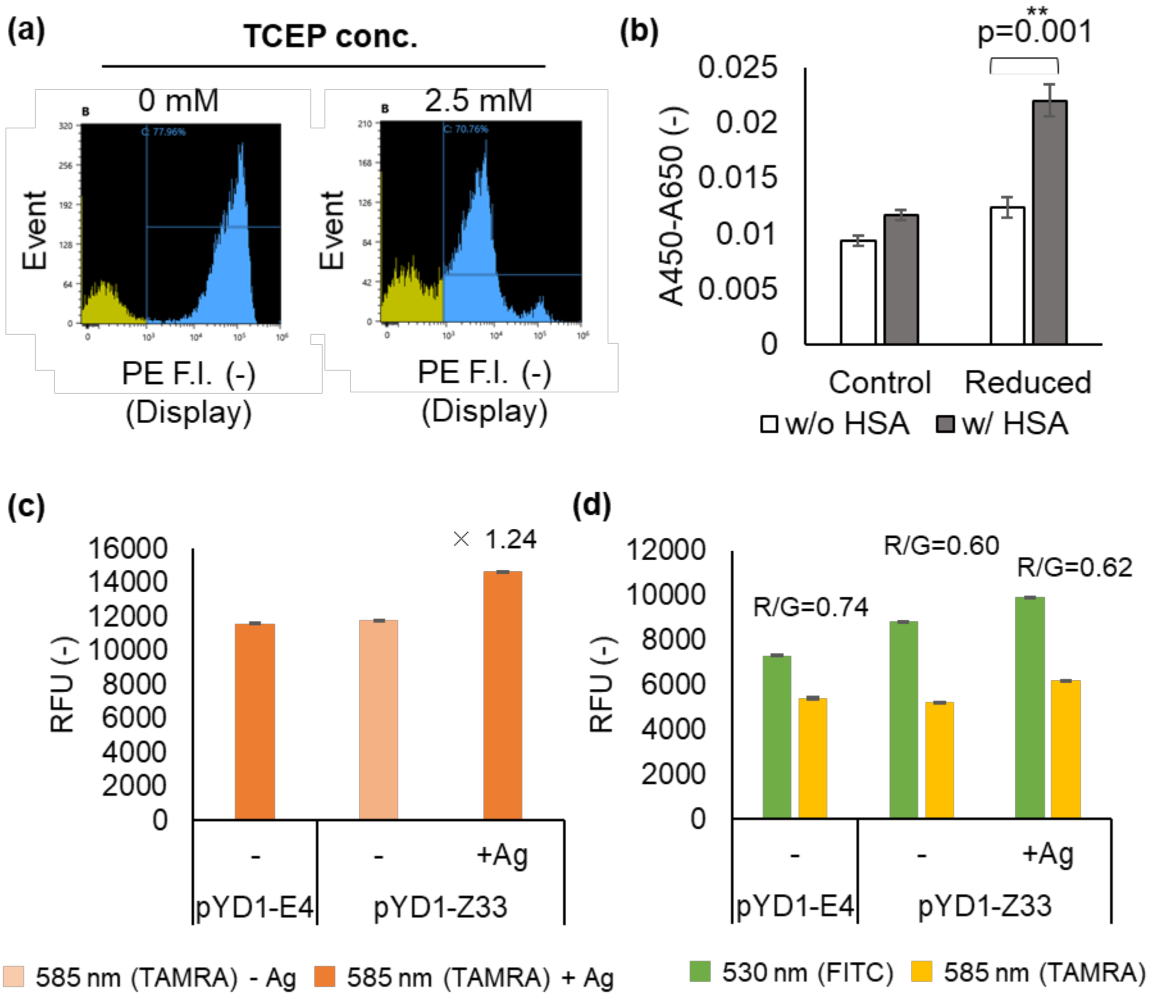

Figure S11. Shaving of anti-HSA nanobody (Z33) displayed on the yeast cell surface. (**a-b**) Yeast-displayed Z33 (≈5.0×10^7 cells) was reduced by 2.5 mM TCEP **(a)** Flow cytometric analysis of reduced yeast cell labeled with PE-conjugated anti-FLAG IgG (Miltenyi Biotec) **(b)** ELISA analysis of reduced Z33. **(c-d)** Fluorescence response analysis of reduced Z33 labeled with FITC-K4-TAMRA. **(c)** Fluorescence of TAMRA (585 nm) exited at 535 nm **(d)** Fluorescence of FITC (530 nm) and TAMRA (585 nm) exited at 483 nm. Error bars represent ±1 standard deviation (SD) (n =3)

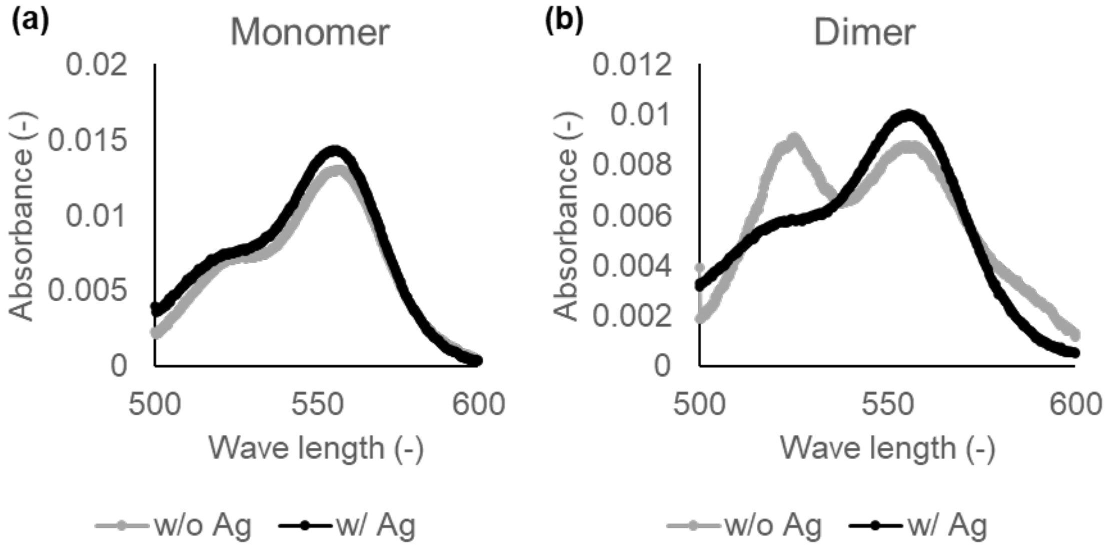

Figure S12. UV spectra of dimerized 5-TAMRA C6-labeled Z33. **(a)** 5-TAMRA C6-labeled Z33 without anti-FLAG M2 IgG **(b)** 5-TAMRA C6-labeled Z33 with anti-FLAG M2 IgG. The gray and black spectrum represents without and with 10 µM HSA, respectively.
